# Why men trophy hunt

**DOI:** 10.1098/rsbl.2016.0909

**Published:** 2017-03-29

**Authors:** Chris T. Darimont, Brian F. Codding, Kristen Hawkes

**Affiliations:** 1 Department of Geography, University of Victoria, Box 1700, Stn CSC, Victoria, British Columbia, Canada V8 W 2Y2; 2 Raincoast Conservation Foundation, General Delivery, Denny Island, British Columbia, Canada V0T 1B0; 3 Hakai Institute, Box 309, Heriot Bay, British Columbia, Canada V0P 1H0; 4 Department of Anthropology, University of Utah, 270 S. 1400 E., Salt Lake City, UT 84112, USA; 5 Global Change and Sustainability Center, University of Utah, 257 S. 1400 E., Salt Lake City, UT 84112, USA

**Keywords:** carnivore, costly signalling, exploitation, Internet, size-selective harvesting

## 1. Introduction

The killing of Cecil the lion (*Panthera leo*) ignited enduring and increasingly global discussion about trophy hunting [1]. Yet, policy debate about its benefits and costs (e.g. [2,3]) focuses only on the hunted species and biodiversity, not the unique behaviour of hunters. Some contemporary recreational hunters from the developed world behave curiously, commonly targeting ‘trophies’: individuals within populations with large body or ornament size, as well as rare and/or inedible species, like carnivores [4]. Although contemporary hunters have been classified according to implied motivation (i.e. for meat, recreation, trophy or population control, [5,6]) as well the ‘multiple satisfactions’ they seek while hunting (affiliation, appreciation, achievement; [7], an evolutionary explanation of the motivation underlying trophy hunting (and big-game fishing) has never been pursued. Too costly (difficult, dangerous) a behaviour to be common among other vertebrate predators, we postulate that trophy hunting is in fact motivated by the costs hunters accept. We build on empirical and theoretical contributions from evolutionary anthropology to hypothesize that signalling these costs to others is key to understanding, and perhaps influencing, this otherwise perplexing activity.

## 2. Man the show off?

Subsistence hunting among traditional ‘hunter–gatherers’, which also targets larger-bodied prey, provides a starting point for understanding trophy hunters from the developed world. Owing to disagreement over the relative importance of potential benefits men receive from hunting, however, evolutionary explanations as to why subsistence hunters target large prey attract competing theories and significant controversy. Some assert that energetic and nutritional returns to hunters and individuals they provision best explain why men accept the costs of big-game hunting (e.g. [8,9]). Others invoke the pressure to share large prey as an explanation for wide distribution of meat (e.g. [10]). But why target prey that will be mostly consumed by others? An alternative hypothesis, consistent with data across hunter–gatherer systems, starts by noting that men generally target species that are not only large-bodied but also—and, importantly—impose high cost (i.e. high failure risk; [11,12]). The hypothesis considers the carcass not only as food but also a signal of the costs associated with the hunter's accomplishment.

The Meriam peoples of Australia provide a flagship illustration of this association. There, men, women and children collect green turtles (*Chelonia mydas*) when they come ashore to lay eggs. In contrast, only men hunt them at sea. Pursuing turtles in boats, hunters accept significant economic and personal cost, including a dive into dangerous waters [13], despite the fact that most of what they acquire will be consumed by other community members [14,15].

Such seemingly irrational behaviour is resolved by costly signalling theory [16] from which the hypothesis draws. The theory considers the social status and prestige that accrue to successful hunters. The Maasai peoples of eastern Africa themselves describe lion killing as a manhood ritual that awards prestige to the hunter who first spears the animal [17]. Why is status awarded? Simply put, killing large, dangerous, and/or rare prey is difficult with high failure risks that impose costs on the hunter. Accordingly, successful hunts signal underlying qualities to rivals and potential allies. This holds true for successful Meriam turtle hunters, who gain social recognition, get married earlier to higher-quality mates, and have more surviving children [14]. For such behaviour to be maintained, even the attempted hunt must signal that the hunter can sustain the handicap of high-cost, low-consumption activity, providing honest evidence of underlying phenotypic quality [14,15,16].

**Figure 1.**
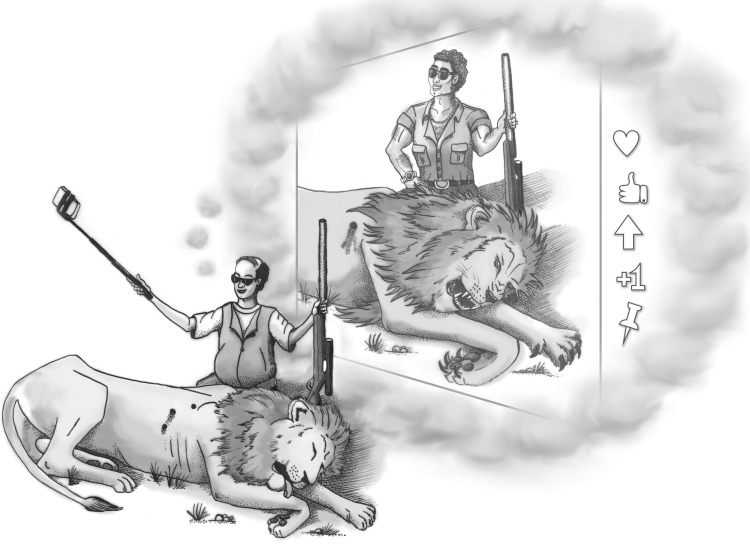
Social media provides some trophy hunters a vast audience to which to signal an ability to absorb the costs of trophy hunting.

We propose that an assessment of contemporary trophy hunting behaviour offers fresh additional evidence for a costly signalling model to explain any big-game hunting. First, inedible species, like carnivores commonly targeted by trophy hunters, make nutritional and sharing hypotheses implausible. Second, evidence for show-off behaviour appears clear. Trophy hunters commonly pose for photographs with their prey, with the heads, hides and ornamentation prepared for display [18]. Interestingly, similar costly display occurs in other taxa. For example, chimpanzees (*Pan troglodytes*) likewise pay a cost in time and effort spent hunting without commensurate food consumption gains; interpretations of related display behaviour support a social status model (*reviewed in* [19]). Similarly, some seabirds like the pigeon guillemot (*Cepphus columba*) show off ‘display fish’, sometimes for hours. Often discarding them, the behaviour is likewise thought to be social, related to site-ownership display [20]. Third, whereas some might argue that caloric returns for edible trophy hunted species are high and associated costs of failure low (owing to advanced killing technology and foods easily purchased by participants), the behaviour still imposes costs that guarantee the honesty of the signal; while rarely costly in terms of danger or difficulty, hunts for endangered species can be extraordinarily expensive. Moreover, even the everyday hunter who targets larger individuals within populations pays the opportunity costs of forgoing income-generating activities as well as sustenance lost by passing up smaller, abundant prey. We note that the signal can honestly reflect a hunter's socio-economic standing (and qualities that underlie it) but not necessarily any remarkable physical abilities ([21]; figure 1), given the efficient technology contemporary trophy hunters employ [4].

A signalling model assumes benefits to both signaller and audience, the latter benefiting from the information they can then use in their own ways. It is unclear what specific benefits—other than increased status—might accrue to trophy hunters. Trophy hunting systems do not lend themselves to testing for patterns associated with reproductive success, as in the Meriam example above. Hunting associations (e.g. Boone and Crockett Club, Safari Club International), however, have elaborate scoring systems that award status. We predict that greater status is bestowed upon those killing larger and/or rarer (i.e. costly) animals. Similarly, no detailed data exist on the potential audience, but we suspect hunters would broadcast the signal to friends and family, colleagues and members of hunting associations or social media groups (see below). Survey and/or interview data, commonly collected in the context of wildlife management or research, may be able to clarify audience composition. If we accept that trophy hunting simply provides a vehicle for status-accumulation, such an interpretation is consistent with those related to the purchase and display of luxury objects (e.g. expensive automobiles, clothes and jewellery), long proposed to serve as forms of competitive signalling [22]. Finally, given that women in hunter–gatherer societies overwhelmingly target small, predictable prey compared with men [12], there are now seemingly puzzling examples of female trophy hunters, often prominent media figures and/or professional hunters sponsored by outdoor companies. We speculate that such behaviour, counter to expected gender norms (and their evolution), might allow for increased attention in an increasingly competitive social media and marketing world (below).

## 3. Costly signalling in a global, commercialized world

Worldwide social media creates for trophy hunters a vast audience to which to boast. Signalling the costs of hunting are no longer restricted to carcass displays in small social groups. Men can now communicate an ability to absorb trophy hunting costs not only to their immediate social group but also—with the help of the Internet—to a global audience. Media abound with costly signals. For example, although probably not a representative sample, many hunters post hunting stories and pictures on online discussion forums, commonly emphasizing the size of kills [21]. Advertisements for hunting equipment likewise frequently emphasize a product's efficacy in securing large specimens. In these ways and more, contemporary culture reinforces trophy-seeking behaviour that probably evolved long ago.

## 4. Policy-relevant research

Although some argue that trophy hunting provides a route to conservation, others contend that trophy hunting can pose significant threats to hunted populations. Interacting with our signalling hypothesis, and of acute conservation concern, is how trophy hunting of rare species can propagate a feedback loop toward extinction. Known as the ‘anthropogenic Allee effect’, demand and associated costs increase when otherwise unprofitable rare resources become attractive, thereby speeding up their decline [23].

We call for more research to evaluate quantitatively the conditions that influence trophy hunting motivation. If the signalling hypothesis explains this behaviour, then policies designed to limit the perceived cost of the activity, dampen signal efficacy or both should reduce trophy hunting. Indeed, recent bans by several governments on the importation of lion remains have probably curtailed demand, despite the hunts themselves remaining legal. And how might shame [24] influence motivation? We predict that social media boasting about lion hunting declined following the widespread shaming after Cecil's death during perhaps the largest media coverage ever associated with wildlife [25]. After all, any perceived benefits of signalling are also probably contingent on associated threats to status, something shaming would erode.
